# Significant elevated CXCL14 and decreased IL-39 levels in patients with tuberculosis

**DOI:** 10.1515/biol-2022-0594

**Published:** 2023-05-18

**Authors:** Min Ding, Hong-xu Wang, Si-jia Gao, Xiao-fei Lai, An-long Li, Jia-jia Bao, Felycia-Fernanda Hosyanto, Lei Xu

**Affiliations:** Department of Respiratory, The First Affiliated Hospital of Chongqing Medical University, Chongqing 400016, China; Department of Laboratory Medicine, The First Affiliated Hospital of Chongqing Medical University, Chongqing 400016, China; Pathogenic Biology Department, School of Basic Medicine, Chongqing Medical University, Chongqing 400016, China; Hospital-Acquired Infection Control Department, First People’s Hospital of Jintang County, Chengdu, 610499, China; Department of Clinical Medicine, Chongqing Medical University, Chongqing 400016, China

**Keywords:** IL-39, CXCL14, tuberculosis, *BCG*, cytokine, biomarker

## Abstract

To explore the serum levels of IL-39, CXCL14, and IL-19 in patients with tuberculosis (TB) along with their clinical significances and their concentration changes in macrophages after *Bacille Calmette-Guérin vaccine* (*BCG*) or *Mycobacterium tuberculosis* (*M. tb*) H37Rv stimulation *in vitro*. The serum levels of IL-39, CXCL14, and IL-19 of 38 TB patients, and 20 healthy staff members were measured by enzyme-linked immunosorbent assay. Moreover, the levels of IL-19, CXCL14, and IL-39 in cultured THP-1 macrophages were detected at 12, 24, and 48 h after stimulation with *BCG* or *M. tb* H37Rv strains. It was found the serum level of IL-39 was significantly reduced and CXCL14 was remarkably elevated in TB patients. *In vitro*, at 48 h after stimulation, IL-39 level of cultured THP-1 macrophages in the H37Rv group was significantly lower than that in the *BCG* and control groups, and the CXCL14 level of cultured THP-1 macrophages in the H37Rv stimulation group was remarkably higher than that in the control group. Therefore, IL-39 and CXCL14 may be involved the pathogenesis of TB, and serum IL-39 and CXCL14 could potentially serve as a new biomarker of TB.

## 1 Introduction

Tuberculosis (TB) represents the leading cause of global deaths from one infectious agent before COVID-19 pandemic and has been recognized as a global public health emergency for the past 25 years [1,2]. Despite the numerous headways in TB diagnosis, there is currently no simple, high-detection test to determine the diagnosis of TB.

IL-39 is a newly identified member of the interleukin-12 (IL-12) family and is a heterodimeric cytokine consisting of the p19 and EBi3 subunits [3,4]. The natural IL-39 (p19/Ebi3) complex is present in the supernatant of cultured RAW264.7 macrophage cells and could be secreted by B cells [3,5]. IL-39 may increase the proliferation of cancer cells [6] and contribute to the pathogenesis of systemic lupus erythematosus [3]. So far, the research on IL-39 is mainly in cancer cell lines, while reports of animal models or human experiments are rare.

The chemokine CXCL14 is a highly conserved homeostatic chemokine that is constitutively expressed in epithelial organs and homeostatically expressed in mucosal tissues [7,8]. It has been well acknowledged that CXCL14 plays a critical role in the metastasis of breast cancer [9].

IL-19 is a member of the IL-10 cytokine family [10] and is also a member of the cytokine IL-20 subfamily [11]. Several cytokines from IL-10 family were proved critical for the regulation of host defense against infections, including IL-10, IL-22, IL-24, and IL-26 [10].

Heretofore, there are only few studies on three cytokines in TB. Research suggests that human immunity to *Mycobacterium tuberculosis* (*M. tb*) is crucial for the occurrence and development of TB [12,13]. Therefore, this study aims to understand the levels of these cytokines in patients with TB and the changes of these three cytokines in cultured macrophages stimulated by *Bacille Calmette-Guérin vaccine* (*BCG*) or *M. tb H37Rv* strains, as well as the further analysis of their clinical relationship with TB.

## 2 Materials and methods

### 2.1 Subjects

All TB patients and healthy staff members were recruited at the respiratory department and physical examination center of The First Affiliated Hospital of Chongqing Medical University from 2020 to 2021, respectively. The protocol was approved by the Clinical Research Ethics Committee of the First Affiliated Hospital of Chongqing Medical University. According to the guidelines of the World Health Organization, we divided the patients with TB into active and inactive groups according to the clinical symptoms and signs, bacteriology, pathology, and radiological detection [14]. Active TB is defined by one or more of the following diagnostic methods: (1) identification of cultured *M. tb* from any clinical specimens or bronchoalveolar lavage fluid, sputum, intratracheal aspirates, and pleural fluid; (2) caseous granuloma recorded in histological examination of tissue specimens; (3) PCR detection of *M. tb* DNA; and (4) chest X-ray examination was performed in combination with clinical symptoms. On the other hand, inactive TB is defined as patients with previous TB or abnormally stable imaging results, who has positive result of tuberculin skin test, negative bacteriologic test, and absence of clinical or imaging evidences of current disease. All TB patients received standardized medical history collections and physical examinations. The T-spot test was performed in strict accordance with the manufacturer’s protocol (Oxford Immunotec Ltd., UK). The serum samples of TB patients were collected before the initiation or reinforcement of treatment and during regular hospital visits, respectively. Control sera were obtained from healthy staff members (*n* = 20). All serum samples were preserved at −20°C.


**Informed consent:** Informed consent has been obtained from all individuals included in this study
**Ethical approval:** The research related to human use has been complied with all the relevant national regulations, institutional policies and in accordance with the tenets of the Helsinki Declaration, and has been approved by the Clinical Research Ethics Committee of the First Affiliated Hospital of Chongqing Medical University.

### 2.2 Macrophage cell line

The THP-1 human macrophage strain was obtained from China Center for Type Culture Collection (CCTCC). The cell line was made into 80 mg/ml suspension, stored at 4°C, and diluted 10 times before use. All the operations were completed under the guidance of the third level Biosafety Laboratory of Chongqing Center for Disease Control and Prevention.

### 2.3 Cell culture and *BCG/H37Rv* stimulation

THP-1 macrophages were cultured in 1640 medium (Gibco, USA) with 10% fetal bovine serum (Gibco, USA) at 37°C and 5% CO_2_. The cell pellet was resuspended with cell growth solution, adjusted the density to 2.5 × 10^5^ cells/ml. Phorbol-12-Myristate-13-Acetate (PMA, SIGMA, USA) stock solution (to a final concentration of 5 ng/ml) was added to the culture to stimulate the transformation of THP-1 cells into macrophages, which was then incubated for 24 h. Subsequently, the cell culture supernatant was discarded, the cells were washed twice with D-Hanks, being replaced with new cell growth medium and incubated for another 72 h. The supernatant was then discarded. The cells were washed twice with D-Hanks and being replaced with cell growth medium containing *BCG* and *H37Rv* strains, respectively (MOI = 10:1). After that, the cells were incubated for 2 h and the supernatant was discarded. The cells were washed twice with D-hanks and replaced with new cell growth medium and samples were taken at 12, 24, and 48 h for the detection of IL-19, CXCL14, and IL-39 [14,15].

### 2.4 Measurement of IL-19, IL-39, and CXCL14

The levels of IL-39 (Ruipan, Shanghai, China), CXCL14 (Novus USA), and IL-19 (R&D Systems, Minneapolis, MI, USA) were determined by using enzyme-linked immunosorbent assay (ELISA). The operation processes were performed in strict accordance with the manufacturer’s protocol.

### 2.5 Statistical analysis

The Kruskal–Wallis test was used to assess the clinical features of the TB patients. CXCL14 concentrations did not have a Gaussian distribution; therefore, the Mann–Whitney *U* test was used to analyze the relationship between serum level of CXCL14 and TB classification or T-spot or *BCG* vaccination. For *in vitro* tests, data were expressed as the means and standard deviations of at least three independent experiments. Statistical significance was determined by Student’s *t* test. All data were expressed as mean ± SD. Differences were assumed statistically significant at *p* < 0.05. All analyses were performed using the Statistical Package for the Social Sciences (SPSS) statistical software for Windows, version SPSS 22.0 (SPSS Inc., IL, USA).

## 3 Results

### 3.1 The demographic and clinical characteristics of study subjects

Totally, there were 38 patients with TB and 20 healthy controls in the current study (Table 1). Among the 38 TB patients, 20 of them had active TB and the rest 18 patients had inactive TB. There was no difference between health control (HC) and TB patients in the aspect of age distribution. The proportion of TB patients (65%) from rural areas was higher than that of the control group (50%). The duration of TB was 5.5 (1.5–6) years (median [interquartile range, IQR]) and 3.2 (2.6, 3.9) years (median (IQR)) for inactive and active TB, respectively.

**Table 1:** Clinical features of the different disease severity in TB patients

Clinical features	HC	TB patients		*p* valve*
		Inactive	Active	(Kruskal–Wallis test)
Age, years (median (IQR))	41 (26–59)	36 (25–48)	40 (32–50)	0.166
Rural area, *N* (%)	10 (50%)	11 (61%)	14 (70%)	0.021
Duration, years (median (IQR))	0	5.5 (1.5–6)	3.2 (2.6–3.9)	0.010
*BCG* vaccination rate, *N* (%)	13 (65%)	10 (55.6%)	7 (35%)	0.010
WBC (10^9^/L)	6.5	5.8	5.2	0.062
Neutrophil (%)	68	42	35	0.012
Lymphocyte (%)	30	57	59	0.001
CXCL14 (pg/ml)	25.12 ± 8.8	63.3 ± 31.4	151.1 ± 106.4	0.000
IL-39 (pg/ml)	0.15 ± 0.05	0.038 ± 0.029	0.031 ± 0.034	0.000
T-spot (+), *N* (%)	0	9 (50%)	19 (95%)	0.002
PPD (+), *N* (%)	2 (10%）	12 (66.7%)	20 (100%)	0.020

HC: health control; IQR: interquartile range; WBC: white blood cells; *BCG*: *Bacille Calmette-Guérin vaccine*; T-spot: Tuberculosis γ Interferon release analysis; PPD: pure protein derivative of tuberculin. *Bold values indicate statistical significance, *p* value <0.05.

There was no significant difference in the count of WBC (vs 6.5, *p* = 0.062) between the TB group and the control group, while the percentage of neutrophils (vs 68%, *p* = 0.012) in the TB group was lower than that of the control group, and the percentage of lymphocytes (vs 30%, *p* = 0.001) was significantly higher. The positive rates of T-spot in groups of active TB, inactive TB, and HC were 95, 50, and 0%. Meanwhile, the positive rates of purified protein derivative (PPD) test in these three groups were 100, 66.7, and 10%, respectively.

### 3.2 The serum levels of CXCL14, IL-39, and IL-19 in patients with TB

**Figure 1:**
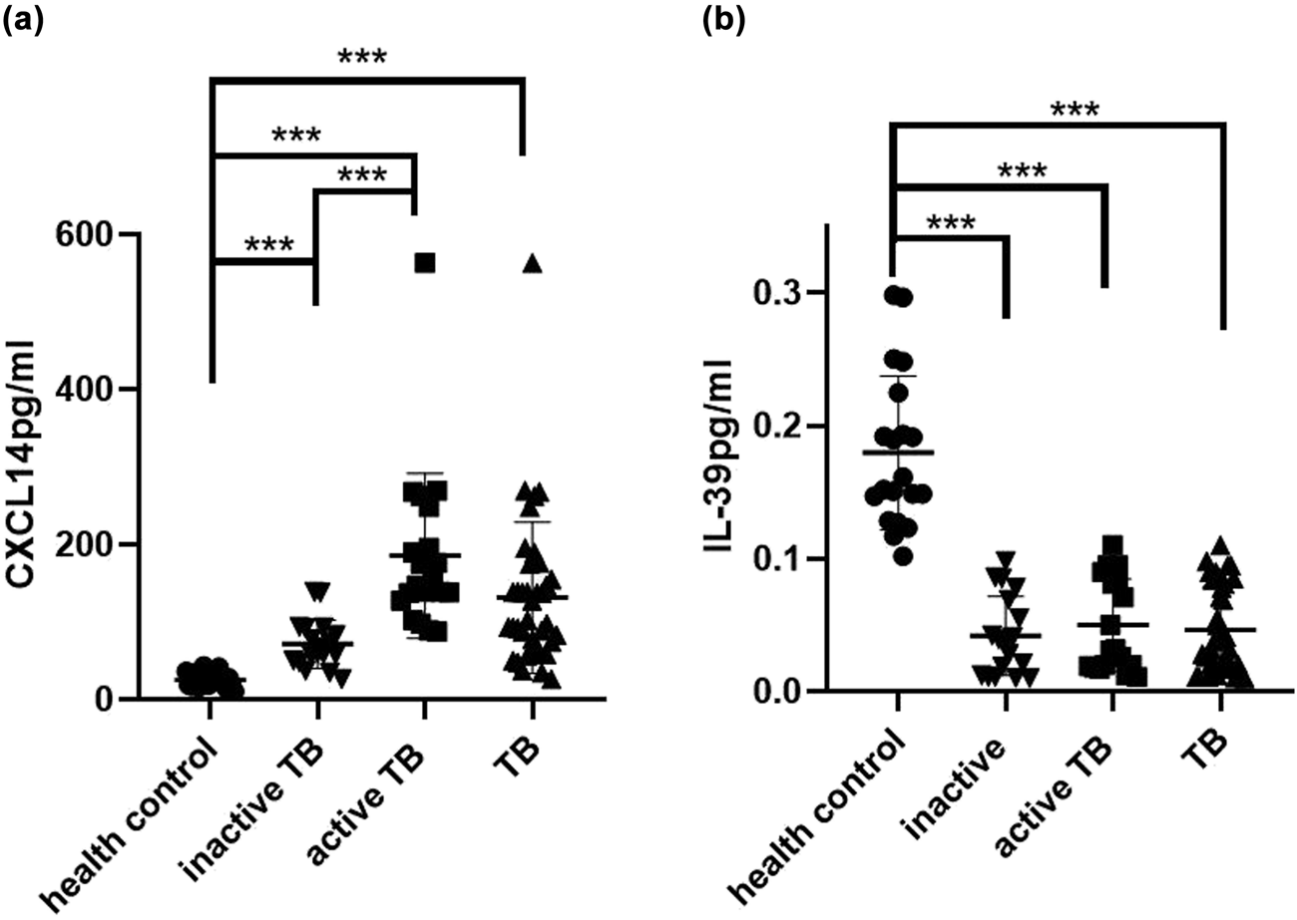
The serum level of IL-39 and CXCL14 in patients with TB. (a) The serum level of CXCL14. (b) The serum level of IL-39. Thirty-eight TB patients (20 active TB patients and 18 inactive TB patients) and 20 healthy controls were recruited in this test. *p < 0.05. **p < 0.01. ***p < 0.001.

Compared with the HC, the serum level of CXCL14 was significantly increased in the TB group and the highest level was in the group of active TB (Figure 1a). The level of IL-39 was significantly decreased in patients with TB (Figure 1b). According to the concentration of CXCL14, patients with active and inactive TB were divided into five groups. The distribution of CXCL14 concentration was statistically different between the active and inactive TB groups. Eighty-five percentage (17/20) of the patients with active TB had CXCL14 >100 pg/ml, while 11.1% (2/18) of the patients with inactive TB had CXCL14 >100 pg/ml, and the concentration of CXCL14 was lower than 150 pg/ml in all patients with inactive TB (Table 2). In addition, there was a statistical difference in the distribution of CXCL14 concentrations between the T-spot-positive and -negative groups in TB patients (Table 3). Moreover, the distribution of CXCL14 level was statistically different between *BCG* vaccination and non-vaccination groups in active TB (Table 4), while there was no statistical difference in IL-19 concentration among these three groups (data were not shown).

**Table 2:** Relationship between serum level of CXCL14 and TB classification

TB	20 ≤ CXCLl4 ≤ 100 pg/ml	100 < CXCLl4 ≤ 150 pg/ml	150 < CXCLl4 ≤ 300 pg/ml	CXCLl4 >300 pg/ml	Test	*Z*	*p* value
Active (*n* = 20)	3 (15%)	7 (35%)	9 (45%)	1 (5%)	Mann–Whitney *U* test	−4.537	0.000
Inactive (*n* = 18)	16 (88.9%)	2 (11.1%)	0 (0%)	0 (0%)			

**Table 3:** Relationship between serum CXCL14 and T-spot in TB patients

T-SPOT	20 ≤ CXCLl4 ≤ 100 pg/ml	100 < CXCLl4 ≤ 150 pg/ml	150 < CXCLl4 ≤ 500 pg/ml	Test	*Z*	*p* value
P (*n* = 28)	10 (26.3%)	8 (21.1%)	10 (26.3%)	Mann–Whitney *U* test	−2.923	0.003
N (*n* = 10)	9 (23.7%)	1 (2.6%)	0 (0%)			

P: positive result; N: negative result.

**Table 4:** Relationship between serum CXCL14 and *BCG* vaccination in patients with active and inactive TB

TB activity	*BCG*	20 ≤ CXCLl4 ≤ 100 pg/ml	100 < CXCLl4 ≤ 150 pg/ml	150 < CXCLl4 ≤ 500 pg/ml	Test	*Z*	*p* value
Active	Y (*n* = 7)	3 (15%)	3 (15%)	1 (5%)	Mann–Whitney *U* test	−1.712	0.002
	N (*n* = 13)	0 (0%)	4 (20%)	9 (45%)			
Inactive	Y (*n* = 10)	10 (55.5%)	0 (0%)	0 (0%)		1.630	0.103/0.183*
	N (*n* = 8)	6 (33.3%)	2 (11.1%)	0 (0%)			

*The *p* value calculated by the chi-square test for ignoring the 150 < CXCLL4 ≤ 500 pg/ml group.

### 3.3 The concentration variation in CXCL14, IL-39, and IL-19 in macrophage culture stimulated by *BCG* or *H37Rv*

**Figure 2:**
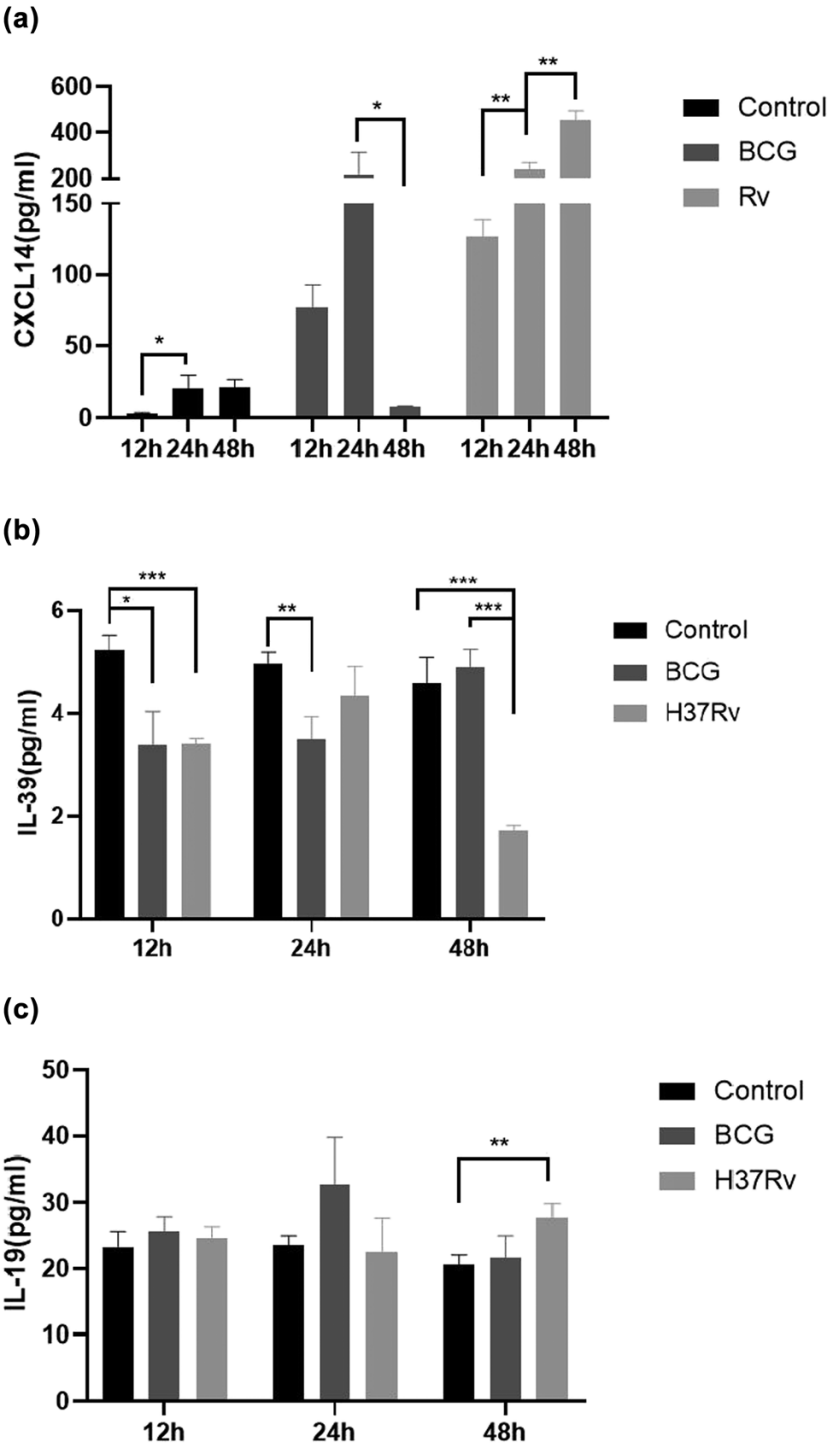
The concentration of IL-39, CXCL14, and IL-19 variation in macrophages stimulated by *BCG* and *H37Rv*. (a) The level of CXCL14 variation in macrophages stimulated by *BCG* and *H37Rv*. (b) The level of IL-39 variation in macrophages stimulated by *BCG* and *H37Rv*. (c) The level of IL-19 variation in macrophages stimulated by *BCG* and *H37Rv*. THP-1 macrophages (2.5 × 10^5^ cells/well) were stimulated by 5 ng/ml PMA first and then were infected with *BCG* or *Mycobacterium tuberculosis H37Rv* (MOI = 10:1), the supernatant was collected at 12, 24, and 48 h, respectively, for cytokines detection by ELISA assay. Results are expressed as the arithmetic mean ± SD from three independent experiments. *p < 0.05. **p < 0.01. ***p < 0.001. And representative experiment as shown.

Regarding the *in vitro* experiments of macrophages, CXCL14 gradually increased within 24 h and then decreased significantly after 48 h in the group of *BCG* stimulation. However, it gradually increased over time in the group of *H37Rv* stimulation (Figure 2a). Variation in IL-39 showed different trends after *BCG* and *H37Rv* stimulation. IL-39 was significantly reduced in macrophages 12 h after *BCG* or *H37Rv* stimulation. After that, the concentration of IL-39 in the group of *BCG* stimulation gradually increased at 24 h and reached the level of the control group at 48 h. Although the IL-39 concentration in the *H37Rv* stimulation group rapidly recovered to the control level within 24 h, it decreased significantly again at 48 h (Figure 2b). There was no statistical difference in IL-19 level *in vitro* test (Figure 2c).

### 3.4 The utility of CXCL14 as biomarkers *versus* T-spot assay for *M. tb* infection

**Figure 3:**
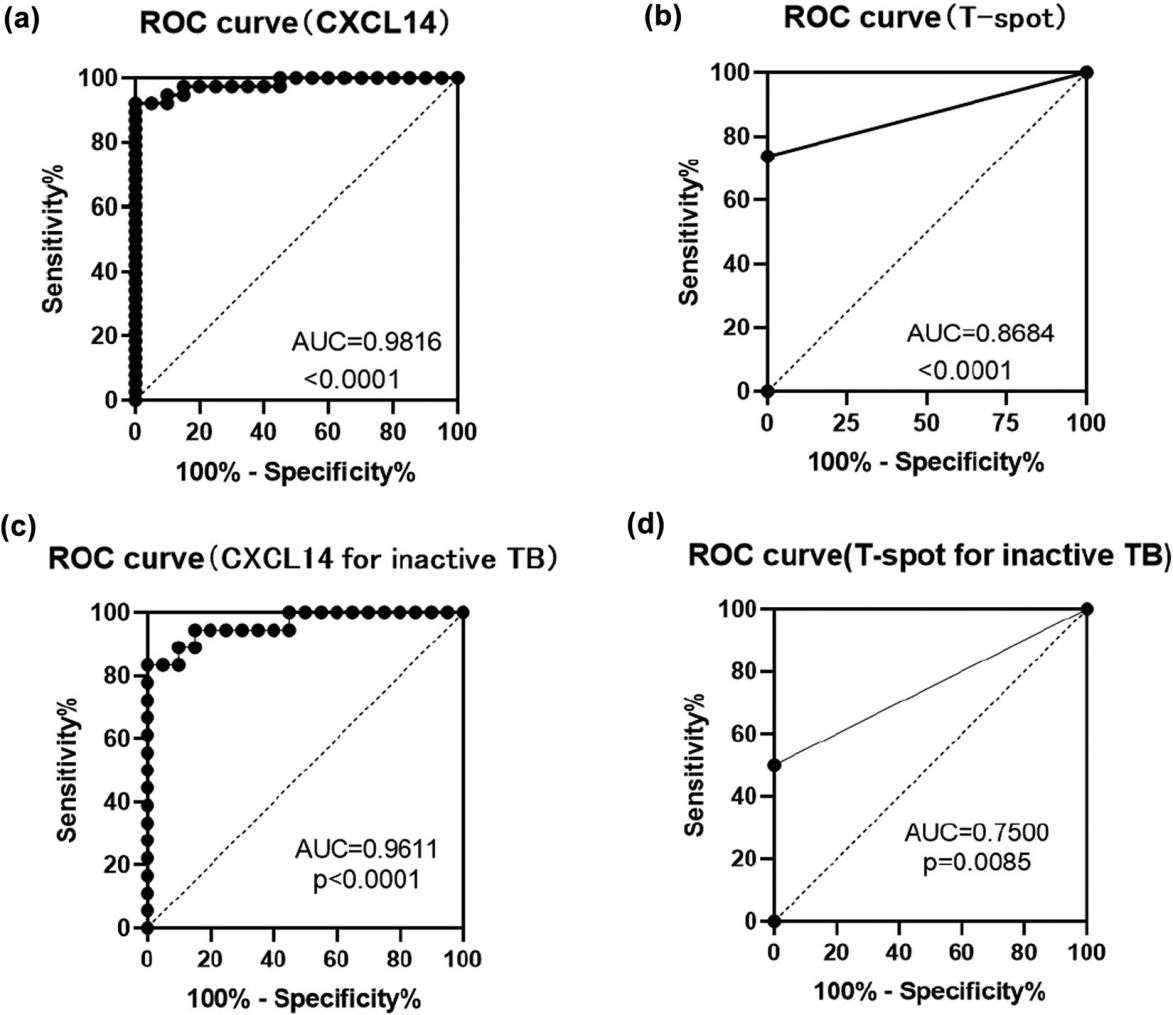
ROC curves for CXCL14 and T-spot in TB patients and inactive TB patients. (a) ROC curves for CXCL14 in TB patients (including inactive TB and active TB patients). (b) ROC curves for T-spot in TB patients (including inactive TB and active TB patients). (c) ROC curves for CXCL14 in inactive TB patients. (d) ROC curves for T-spot in inactive TB patients.

To determine whether CXCL14 could be the biomarkers or diagnostic tests in TB, we calculated the receiver operating characteristics (ROC) curves for CXCL14 and these data were compared with the T-spot assay, an ideal method for TB diagnosis at present. The AUC of CXCL14 as diagnosis biomarkers for TB and inactive TB was 0.9816 (*p* < 0.0001, sensitivity: 92.11%, specificity: 95%) and 0.9611 (*p* < 0.0001, sensitivity: 83.33%, specificity: 95%), compared with the results test of T-spot assay, which was 0.8684 (*p* < 0.0001, sensitivity: 73.68%, specificity: 100%) and 0.7500 (*p* = 0.0085, sensitivity: 50%, specificity: 100%), respectively (Figure 3). These results showed that CXCL14 could be a possible molecular marker to screen serum samples with *M. tb* infection.

## 4 Discussion

TB remains a major global health problem and represents the leading global cause of death from an infectious agent before COVID-19 pandemic [2,13]. Controlling the spread of TB requires a vaccine that can prevent TB. However, due to the population who received *BCG* vaccination, the PPD test results are positive, and they are also positive in individuals with *M. tb* infection. Thereby, doctors need to differentiate *M. tb.*-infected individuals from *BCG*-vaccinated populations [16]. Meanwhile, the body’s defense status against *M. tb* fluctuated with time and condition. Therefore, it is of great significance to continuously explore the ideal markers associated with TB infection.

In the current study, we have performed the first evaluation of serum levels of IL-39, CXCL14, and IL-19 in patients with TB. Also, we tried to explore the changes in the levels of these cytokines in cultured macrophages stimulated by *BCG* and *M. tb H37Rv*.

IL-39 is a new member of the IL-12 family, in addition to IL-12, IL-23, IL-27, and IL-35. All members of the IL-12 cytokine family consist of two kinds of subunits: an α-cytokine subunit and a β-cytokine subunit [17]. This family is considered to be closely related to the occurrence and development of inflammation, and chain pairing rearrangement (disorder) is an important feature of this family. For instance, IL-12 has an α-subunit (IL-12p35) and a β-subunit (IL-12p40) [18]. IL-23 is strongly mediated through its two subunits (IL-23p19 and IL-12p40). IL-27 is made of with IL-27p28 and Epstein–Barr virus-induced gene3 (EBi3) [18]. IL-35 shares the IL-12p35 subunit with IL-12 and EBi3 with IL-27. Although the members of the family are composed of some common subunits, their functions are different. For example, IL-12 recognizes its receptors IL-12Rβ1 and IL-12Rβ2. It leads to the binding of non-receptor tyrosine protein kinase (Tyk2) and Janus kinase 2 (JAK2), activation of STAT4, and stimulation of IFN- γ. It can inhibit the proliferation of HIV. IL-23 passes through IFN-α to inhibit HIV. IL-27 cooperates with JAK2 to phosphorylate stat1–stat3 dimer through Jak1 and IFN-λ1 to inhibit HIV and HBV. Moreover, IL-35 inhibits IFN-γ and TNF-α and promotes the proliferation of HBV [19]. Even though IL-39 shares IL-23p19 with IL-23 and shares EBi3 [3] with IL-27 and IL-35, their roles in human infectious diseases are unclear. It has not been determined whether IL-39 is an inflammatory cytokine or not. On the other hand, the natural IL-39 (p19/EBI3) complex is present in the supernatant of cultured RAW264.7 macrophages and can be secreted by B cells [3,5]. Recently, IL-39 was considered a pathogenic factor in acute Graft-vs.-Host Disease (GVHD) [17], as its serum level in the recipient was positively correlated with the occurrence of acute GVHD [20]. Interestingly, we discovered the reduction in serum level of IL-39 in TB patients, and patients with active TB had the lowest level of IL-39. Meanwhile, after 48 h of stimulation, IL-39 level of THP-1 macrophages in the *H37Rv* stimulation group was significantly lower than that in the *BCG* and control groups. It may suggest that IL-39 could serve as a factor in the pathogenesis of TB and would shed light on further researches of IL-39 in TB. On the other hand, previous study suggested that IL-39 could be a theoretical cytokine in human, and it is secreted below the lower quantity of detection or it has no functional role [21]. In this study, IL-39 was detected by a commercially available ELISA reagent. Thereby, it is necessary to further study the role of IL-39 in TB infection.

Secondly, we found that CXCL14 levels in the serum of TB patients were increased, especially in the active TB group. After 48 h of stimulation, the level of CXCL14 in THP-1 macrophages in the *H37Rv*-stimulated group was significantly higher than that in the control, while that in the *BCG*-stimulated group was significantly lower than that in the control group. Thus, an elevation of CXCL14 may indicate TB infection. In addition, there were statistical differences in the distribution of CXCL14 levels between the control and active TB groups that were *BCG* vaccinated and non-vaccinated, and between active and inactive TB groups, as well as TB patients with T-spot-positive results and those of negative results. Collectively, our study suggests, for the first time, that elevated CXCL14 may be associated with TB infection, which is consistent with T-spot results, and may be used to distinguish between active and inactive TB that is independent of *BCG* vaccination. Although the potential role of CXCL14 in cancer has been a focus of research in recent years [9,22], other aspects of CXCL14 are also being explored. CXCL14 also regulates platelet function and migration [23,24] and mediates the recruitment of CD14 monocytes in the skin [25]. Furthermore, there was a study showing that CXCL14 shows certain antibacterial activity, and the antibacterial efficacy of CXCL14 is more advantageous than known anti-microbial peptides like human β-defensin-2 or chemokine CCL20 [25,26]. The principle may be that CXCL14 effectively kills *Pseudomonas aeruginosa*, *Streptococcus pyogenes*, and *Streptococcus pneumoniae* in a dose-dependent manner by inducing membrane depolarization and membrane rupture [26]. Furthermore, CXCL14 has been shown to have broad antibacterial effects against skin Gram-positive bacteria and *Candida albicans*, and those antibacterial activities exhibited by human and mouse CXCL14 can be neutralized by anti-CXCL14 monoclonal antibodies [27]. Thus, we speculate that the increase of CXCL14 in the peripheral blood of TB patients may be an immune response of the host against *M. tb*. It remains uncertain whether CXCL14 plays a protective role in TB infection, but our study provides some interesting clues.

Third, IL-19 is a member of the IL-10 cytokine family [10] and is also a member of the cytokine IL-20 subfamily [11].

Circulating IL-19 levels in patients with tuberculous lymphadenitis were significantly lower than those in TB and healthy controls, and their circulating IL-19 levels increased significantly after completion of anti-tuberculous treatment [27]. However, we found no significant differences in IL-19 levels among the active and inactive TB groups, as well as the control. Furthermore, there was no statistical difference in IL-19 level of THP-1 macrophages cultured *in vitro* after *BCG* or *H37Rv* stimulation.

Currently, the main laboratory detection methods of *M. tb* infection include acid fast staining, TB antibody detection, and real-time quantitative PCR. Unfortunately, these methods have numerous disadvantages, such as unsatisfactory sensitivity and specificity, high sample requirements, and sensitivity to immune status. A new method of T-spot TB assay (interferon [IFN]-γ release analysis) based on the detection of IFN secreted in *M. tb*-specific T cells stimulated by *M. tb*-specific antigens has been successfully used in determine the presence of *M. tb* infection. In this study, the T-spot assay is used as a comparison method. These ROC curves showed better sensitivity and similar specificity for CXCL14 in the serum, which are used as auxiliary diagnostic tests for TB including inactive TB and active TB. Therefore, serum CXCL14 may be combined with T-spot as a biomarker for screening for TB infection.

To sum up, IL-39 could serve as a protective factor in the pathogenesis of TB and CXCL14 may play a role in TB infection, which provided some interesting clues on further researches of IL-39 and CXCL14 in TB. More importantly, the cytokines show a greater predicate value for the diagnosis of TB and the evaluation of disease activity. Altogether, the recent study suggests that chemokines, produced by immune cells and induced by other molecules, occupy a vital role in the pathogenesis of TB through multifarious pathways.
